# Eye-tracking-derived foveal biomarkers and functional alterations in dry AMD: findings from a controlled clinical study

**DOI:** 10.1186/s40942-026-00838-x

**Published:** 2026-03-27

**Authors:** Bjørn André Helland-Hansen, Alexander Sverstad, Goran Petrovski, Stig Einride Larsen

**Affiliations:** 1 Centre for Eye Research and Innovative Diagnostics, Department of Ophthalmology, Oslo University Hospital and University of Oslo, Oslo, 0424 Norway; 2 Bulbitech AS, Floor 3, Dybdahls veg 5, Trondheim, 7051 Norway; 3 Department of Ophthalmology, Vestfold Hospital Trust, Tønsberg, Norway; 4 Department of Ophthalmology, University of Split School of Medicine and University Hospital Centre, Split, 21000 Croatia; 5 UKLONetwork, University St. Kliment Ohridski-Bitola, Bitola, 7000 North Macedonia; 6 Meddoc Research, Hvamstubben 14, Skjetten, 2013 Norway

**Keywords:** Age-related macular degeneration (AMD), Agreement Index (AI), Contrast sensitivity, Dynamic visual testing, Foveal adaptation, Foveal function, Intraclass Correlation Coefficient (ICC), Luminance adaptation, Retinal biomarkers, Stability Index (SI), Visual acuity

## Abstract

**Purpose:**

Age-related macular degeneration (AMD) is the leading cause of vision loss in the elderly, and early detection is essential for timely intervention and slowing disease progression. This study validated novel foveal function tests using the Bulbicam (BCAM) device for differentiating early and intermediate dry AMD from healthy controls (HCs) and to identify functional variables that may serve as candidate biomarkers.

**Methods:**

In a controlled clinical study, 17 patients with early and intermediate dry AMD and 17 age-matched HCs were assessed using the proprietary Bulbitech “ACOLAPT” (Acuity, Contrast, Light Adaptation) test. Each eye was classified as either the “Worst eye” or “Best eye” for AMD patients based on predefined clinical criteria. Foveal function metrics were derived from the five main variables: Pursuit acuity (PuAc), Pursuit contrast sensitivity (PuCS), Strobe Pursuit Contrast Sensitivity(StCS), (PuCS − StCS) / PuCS (ΔCS), and Variable Strobe Pursuit Object Detection(VaSt). Test validity, reliability, repeatability, and stability were assessed. Validation of ACOLAPT included Analysis of Variance (ANOVA) and Receiver Operating Characteristic (ROC) analyses to assess discriminative capacity, intraclass correlation coefficients (ICC) and Bland-Altman plots with Agreement Index (AI) to evaluate reliability and Stability Index (SI) for estimation of stability.

**Results:**

All five BCAM tests showed significant differences between AMD patients and HCs in the worst eye (*p* ≤ 0.05). PuAc and PuCS showed good reliability (ICC ≥ 0.77), good repeatability (AI ≥ 0.75), and excellent stability (SI ≥ 0.59). The novel StCS test demonstrated excellent reliability (ICC = 0.96), excellent repeatability (AI = 0.88), and excellent stability (SI = 0.58). Despite good ICC values, VaSt and ΔCS had lower repeatability (AI ≤ 0.25), highlighting the importance of AI and SI metrics.

**Conclusion:**

PuAc, PuCS, and StCS fulfilled the predefined criteria for reliability, repeatability, and stability, supporting their use as candidate biomarkers at both population and individual patient levels. VaSt also met the predefined biomarker criteria through preserved cross-session stability despite lower single-trial agreement. ACOLAPT’s ability to assess multiple aspects of foveal function, along with enhanced stability and repeatability metrics, suggests that it could be valuable in clinical settings for early AMD detection and monitoring.

**Supplementary Information:**

The online version contains supplementary material available at 10.1186/s40942-026-00838-x.

## Introduction

Age-related macular degeneration (AMD) is the leading cause of irreversible central vision loss in older adults and is rising in prevalence as populations age [1, 2]. The structural course of AMD is well described. Drusen accumulate, the retinal pigment epithelium (RPE) deteriorates, and the choriocapillaris narrows [3]. Furthermore, photoreceptor density falls, synaptic architecture remodels, and microglia increase in the outer plexiform layer [4]. These changes are believed to begin long before standard clinical features deviate from the normal range. Taken together, these early structural events contrast sharply with the tools that clinicians currently rely upon.

Routine assessment relies on structural imaging and high-contrast charts. OCT is sensitive to drusen height and local thinning, yet mainly detects later metabolic consequences [5]. Photoreceptors and synapses show degeneration in eyes with drusen, even when vision is still normal [6]. High-contrast acuity often remains near normal until advanced disease [7]. Conventional contrast charts probe thresholds under steady viewing, where sensitivity is shaped by the gain and noise properties of post-receptoral pathways once the cones have reached their adaptation state [8–11]. As a result, early temporal disturbances remain largely unrecorded by standard static tests. Patients may notice functional issues such as fluctuating clarity, slower reading, or delayed recovery after bright scenes long before structural criteria are met. Several studies confirm these early complaints despite normal acuity and OCT findings, describing reduced temporal stability and delayed recovery after glare in early AMD [12].

These early complaints arise in the cone-driven fovea, where sensitivity must be adjusted from moment to moment as luminance changes [13]. When this rapid adjustment becomes less efficient, symptoms reflect timing rather than spatial resolution. Static tools may fail to capture early disturbance.

Classical functional tests provide only partial coverage of this domain. Dark-adaptation protocols capture rod physiology but require prolonged recovery [14]. Psychophysical batteries show site-to-site variability and demand extensive participant cooperation [15]. Microperimetry, while providing sensitivity mapping, still uses static targets and thus may miss transient dysfunction [16, 17]. The earliest functional shifts in AMD can pass unmeasured. These limitations leave a clear gap in assessing rapid, cone-driven foveal function.

The ACOLAPT (Acuity, Contrast, Light Adaptation) test family was developed to target this gap. ACOLAPT’s five sub-tests probe dynamic spatial precision, contrast gain, and temporal recovery using moving or flickering stimuli, potentially revealing subtle functional abnormalities that structural measures (like OCT thickness) and high-contrast vision tests do not detect in early AMD, thus targeting cone adaptation kinetics that static charts or imaging cannot measure. Because functional biomarkers must support both research and clinical follow-up, they need to show reproducible group discrimination and stable within-eye behaviour on repeat testing [18]. With these criteria in view, we designed the present study to evaluate how each ACOLAPT variable behaves under repeated measurement.

While the molecular mechanisms underlying phototransduction are well characterised, including the kinetics of amplification and adaptation at the photoreceptor level [19], the present work focuses on the measurable behavioural consequences of these processes in the intact visual system.

Here we assess five ACOLAPT variables as candidate diagnostic, staging, and monitoring biomarkers for early and intermediate dry AMD. We assess their discriminatory performance and evaluate their reliability using complementary indices: the Intraclass Correlation Coefficient (ICC) for population-level reproducibility, the Agreement Index (AI) for within-eye repeatability, and the Stability Index (SI) for cross-session steadiness. Together these indices distinguish variables suited for research from those appropriate for individual monitoring. Our hypothesis was that dynamic foveal function would reveal abnormalities even when OCT parameters and standard chart measures remain within normal limits.

## Methods

Patients with early or intermediate dry AMD were recruited in the Oslo area through the outpatient ophthalmology clinic at Oslo University Hospital. Recruitment was conducted through clinic-based information material and supplementary announcements distributed via relevant patient organisations and social media channels. Interested individuals contacted the study team and were screeEned for eligibility according to predefined inclusion and exclusion criteria.

The AMD and control groups were closely matched for age and sex. The mean age in the AMD group (*n* = 17) was 67.9 years (SD 9.6; range 49.6–80.2), compared with 66.0 years (SD 10.5; range 48.1–84.5) in healthy controls (*n* = 17). Women constituted 64.7% of the AMD group (11/17) and 58.8% of controls (10/17), with men accounting for 35.3% and 41.2%, respectively. Among participants with AMD, the mean self-reported duration of disease was 6.1 years (SD 5.1; range 0.2–15.9).

This controlled, parallel-group study compared dynamic foveal function in patients with early or intermediate dry AMD and age-matched healthy controls. The groups differ inherently in disease status, so randomisation was not applicable. The purpose was twofold. First, to determine whether ACOLAPT variables separate patients from healthy ageing at the population level. Second, to examine the repeatability and stability of each variable within the same eye to judge its suitability as a patient-level biomarker.

To estimate within-eye repeatability with sufficient precision, AMD participants completed six repeated sessions, while controls completed two. This asymmetry increased the range of intra-individual variance in the disease group without compromising between-group comparison. Both eyes were included for all participants. Eyes were later classified as best or worst according to the Modified International Classification (NICE-preferred) [20] after all data had been collected. If this classification graded both eyes identically, best and worst eye was decided by visual acuity. This post-hoc classification avoided bias during test administration and preserved the independence of measurement.

All procedures followed local institutional guidelines. The Bulbicam (BCAM) device used in this study is CE-marked, and participants gave written informed consent.

### Device and testing environment

Data were recorded using the BCAM system (Bulbitech AS, Trondheim, Norway). The device combines two independently controllable displays with a single infrared camera using dark-pupil, bright-pupil, and corneal-reflex tracking at 400 frames per second. Cold mirrors create a fused virtual image straight ahead while allowing infrared light to pass to the camera. The system is stationary on a desk mount, allowing stable head positioning utilising a chin and forehead rest.

Testing took place in a quiet, moderately lit room. BulbiHub software recorded interpupillary distance and refraction, and corrective lenses were inserted to ensure relaxed viewing without accommodation. Setup required two to three minutes. Once the device had been adapted, participants viewed stimuli solely from the device screens.

### Clinical examination

All participants underwent standard ophthalmic assessment. Best-corrected visual acuity (BCVA) was measured using a logMAR ETDRS chart. Contrast sensitivity (CS) was assessed using a Pelli–Robson chart. Intraocular pressure was recorded, and multimodal imaging included OCT (RS-3000, NIDEK Co.), fundus photography (Optos California), and slit-lamp biomicroscopy.

This clinical profile allowed best- versus worst-eye classification and provided reference values for structural comparison.

### ACOLAPT procedure

The ACOLAPT battery is based on a pursuit-failure paradigm. As a moving stimulus becomes progressively more difficult to detect through systematic changes in size, contrast, or strobe frequency, gaze is continuously tracked using corneal reflection and pupil-centre signals. The system identifies the point at which smooth pursuit can no longer be maintained. When pursuit fails, a brief contracting red guide re-centres fixation before the stimulus reappears at a slightly easier level. Thresholds are determined using an adaptive staircase: pursuit failure triggers a step toward an easier level, whereas sustained pursuit results in a more demanding level. After multiple reversals, the staircase converges to a pursuit-based threshold without operator involvement.

Each subtest lasts approximately 40 s, with total examination time around five minutes once participants are familiar with the task. Testing is monocular. The fellow eye views a uniform white field to maintain stable pupil size and minimise confounding effects of pupillary fluctuations. Except in the pursuit acuity subtest, the stimulus diameter is 0.29 degrees (17.6 arcminutes). The target follows a linear semi-random trajectory composed of straight segments interspersed with brief oscillatory reversals, ensuring continuous engagement of pursuit mechanisms without predictable tracking.

### Definition of ACOLAPT variables

#### Pursuit acuity (PuAc)

PuAc quantifies dynamic spatial resolution. A stimulus composed of adjacent bright and dark elements moves across a grey background while progressively decreasing in diameter. The smallest diameter that can be tracked defines the threshold. The result is expressed as the log10 of the minimum resolvable stimulus diameter (arcminutes), analogous to logMAR. This metric represents the limit at which fine spatial detail can no longer be resolved under motion.

#### Pursuit contrast sensitivity (PuCS)

PuCS assesses dynamic contrast sensitivity during motion. A fixed-size stimulus composed of adjacent bright and dark elements moves across a grey background whose luminance matches the target’s mean luminance, thereby maintaining a constant average light level throughout testing. Contrast, defined in Weber terms, is progressively reduced in discrete steps while overall luminance remains stable. Because the task requires detection rather than identification, it primarily probes retinal and early post-receptoral contrast gain mechanisms under dynamic conditions. The threshold is defined as the lowest contrast that still permits sustained pursuit. Results are expressed as log10 contrast sensitivity (Pelli–Robson units).

#### Strobe Pursuit Contrast Sensitivity (StCS)

A moving stimulus is viewed under 2 Hz 167 ms flashes. Each pulse suppresses sensitivity; the interval allows partial recovery. The threshold reflects the recovery dynamics of cone-driven gain restoration. The test was otherwise similar to PuCS. Values are expressed in Pelli–Robson equivalents.

#### Delta contrast sensitivity (ΔCS)

ΔCS is a derived index calculated as (PuCS − StCS) / PuCS. It quantifies the proportional reduction in contrast sensitivity induced by flicker. Higher values indicate greater susceptibility to temporal modulation, whereas lower values reflect greater resilience of contrast processing under rhythmic suppression.

#### Variable strobe object detection (VaSt)

VaSt measures temporal endurance of adaptive mechanisms. Target contrast remains constant while the frequency of the global luminance pulse increases in a staircase from 0.5 to 15 Hz. Each pulse lasts 167 milliseconds. As frequency increases, the available recovery interval shortens. The threshold is defined as the highest frequency at which pursuit can be maintained. Results are expressed in hertz and represent the temporal limit of functional recalibration under repetitive suppression.

### Statistical approach

The statistical plan followed the framework set out in the Appendix under Biomarker Decision Rules and Statistical Methodology. All analyses distinguished population-level repeatability from patient-level repeatability, and each ACOLAPT variable was judged according to validity, ICC, AI, and SI. Formal thresholds and decision rules are given in Supplement 1 (Biomarker Decision Rules).

#### Overview

Population discrimination between AMD and healthy controls was assessed using analysis of variance and receiver operating characteristic (ROC) curves. Within-eye repeatability and cross-session stability were assessed using three complementary indices: the Intraclass Correlation Coefficient (ICC), the Agreement Index (AI), and the Stability Index (SI).

To guide interpretation, we classified each ACOLAPT variable along two axes. The first concerns repeatability, which reflects whether a measurement captures a physiological process stably and quantitatively. Repeatability appears at two scales: between participants, expressed by ICC, and within the same eye, expressed by AI and SI. A variable that repeats faithfully at one or both scales can be regarded as a dependable index of the underlying visual mechanism.

The second axis concerns validity, which reflects whether the measurement distinguishes healthy ageing from AMD or separates earlier from later functional disturbance. A variable may therefore be physiologically repeatable without being diagnostically useful, or diagnostically valid without meeting the more stringent within-eye repeatability thresholds.

Thresholds and decision rules are specified in Supplement 1 (Biomarker Decision Rules) and are cited where results are interpreted.

#### Validity

A variable was considered valid if either the AMD–control difference was significant or the ROC area under the curve (AUC)-lower 95% confidence bound exceeded 0.50.

#### Population-level repeatability

ICC(3,1), a two-way mixed-effects absolute-agreement model, quantified between-participant repeatability. ICC values greater than 0.50 were considered acceptable.

#### Within-eye repeatability

The Bland–Altman method quantified agreement between repeated measurements. The AI provided a scale-free summary of precision. AI values greater than 0.50 indicated moderate or better repeatability.

#### Temporal stability

The SI compared within- and between-participant variance. SI values greater than 0.14 indicated acceptable stability, with classification thresholds derived from the corresponding F-distribution quantiles, as detailed in Supplement 2 (Statistical Methodology).

#### Classification

Variables meeting the validity criterion and both repeatability scales (ICC and AI, with SI applied where relevant) were classified as reliable according to the Appendix. Variables meeting validity and ICC alone were classified as population biomarkers. Variables meeting validity and within-eye repeatability (AI and SI according to the AI–SI rules) were classified as patient-level biomarkers.

All analyses were performed in SAS 9.04 (Maintenance Release M6). Confidence intervals for ICC were estimated using standard parametric methods, and confidence intervals for SI were obtained by non-parametric bootstrap. Full methodological detail is provided in the Appendix.

## Results

**Fig. 1:**
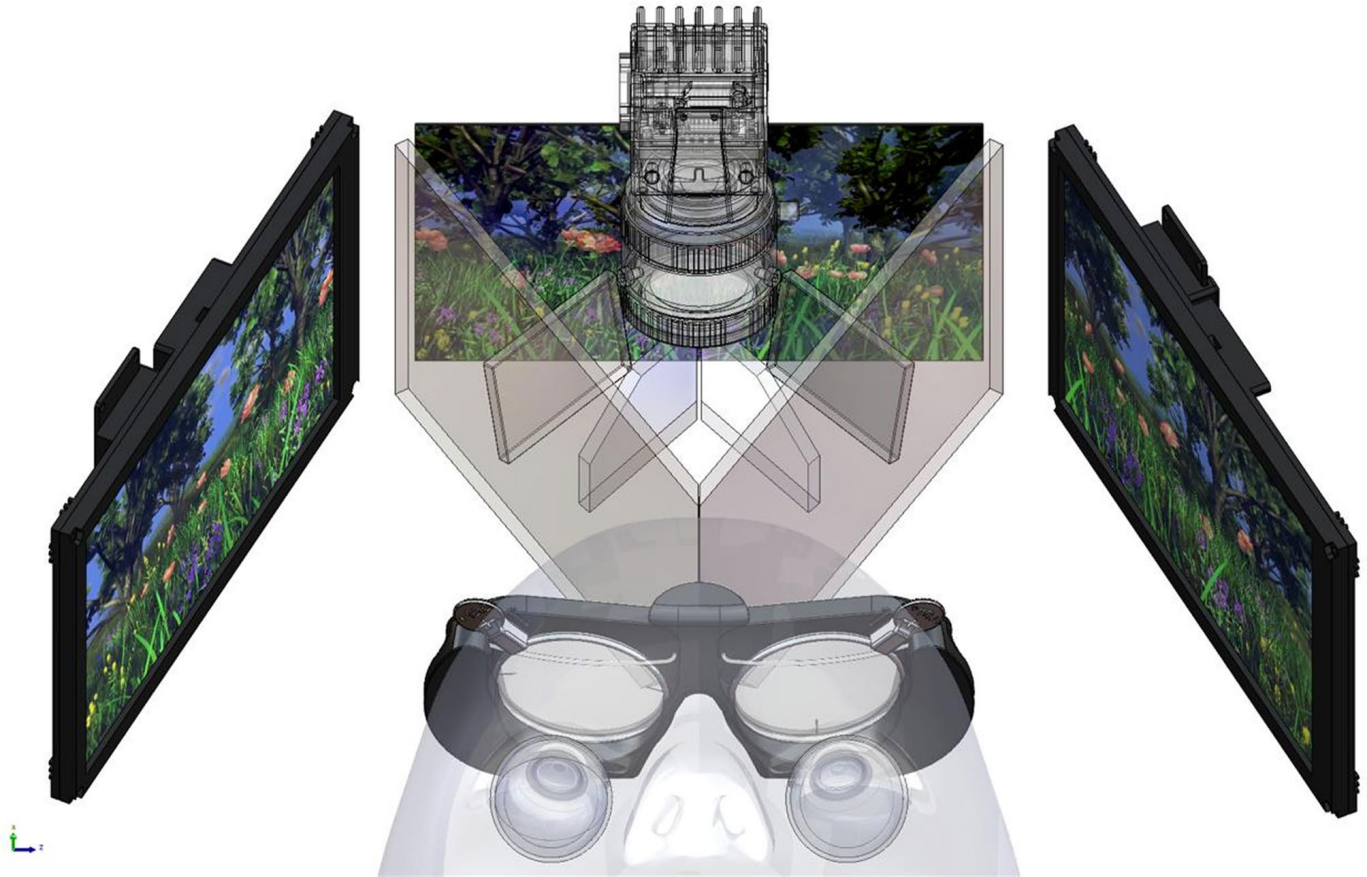
Illustration of Bulbicam architecture. The patient sees the reflection of the displays in the cold mirrors, forming a composite 3D virtual image straight ahead. Cold mirrors let infrared light through, thus enabling the filming of the eyes

Classification of validity, repeatability, and biomarker status followed the pre-specified criteria in Supplement 1 (Biomarker Decision Rules). SI classification thresholds and reliability limits follow Supplement 2 (Statistical Methodology) (Fig. 1).

### Pursuit acuity (PuAc)

#### Worst eye

**Fig. 2:**
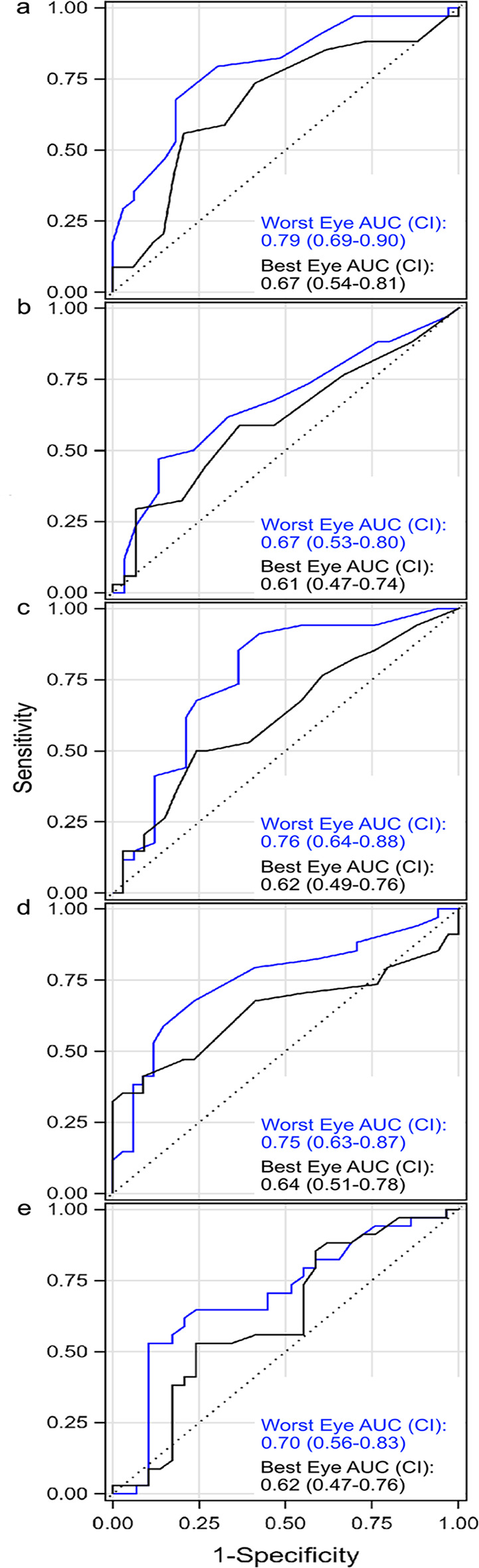
Receiver operating characteristic (ROC) curve of the worst eye (blue line) and the best eye (black line) for **a**) Pursuit acuity (PuAc); **b**) Pursuit contrast sensitivity (PuCS); **c**) Fixed frequency_Variable contrast (StCS); **d**) Variable frequency_Fixed contrast (VaSt), and **e**) ∆CS. AUC: area under the curve

**Fig. 3:**
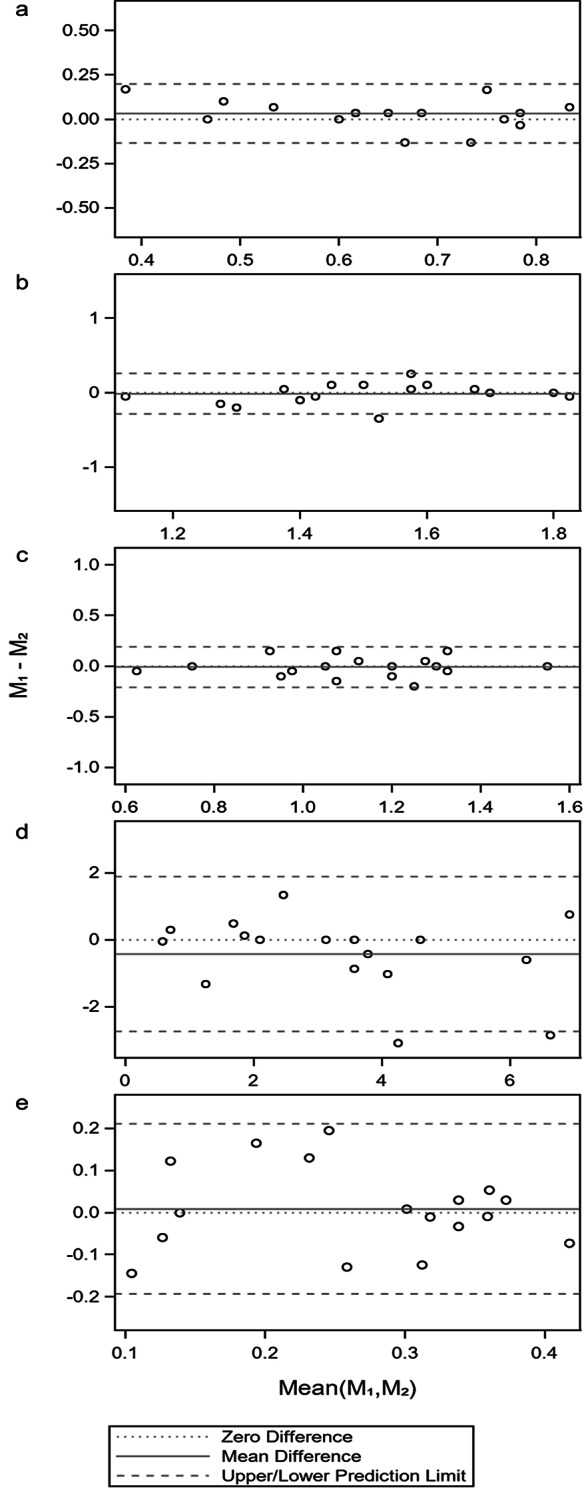
Bland-Altman plot of the worst eye on **a**) Pursuit acuity (PuAc); **b**) Pursuit contrast sensitivity (PuCS); **c**) Fixed frequency_Variable contrast (StCS; **d**) Variable frequency_Fixed contrast (VaSt, and **e**) ∆CS. The mean value of the two observations is given on the x-axis, and the difference between the two observations is on the y-axis. The horizontal dotted lines show the upper- and the lower agreement limits

PuAc was worse (higher) in AMD than in controls (*p* < 0.001; Table 1). The ROC AUC was 0.79 (95% CI 0.69–0.90; Fig. 2a). ICC(3,1) was 0.83 (Table 2). AI was 0.75 (Fig. 3a). SI was 0.56 (95% CI 0.56–0.71; Table 3), classified Excellent. PuAc therefore met validity and both repeatability domains, qualifying as a strong biomarker within this framework.

**Table 1:** Validation of Bulbicam ACOLAPT test variables, comparing patients with age-related macular degeneration (AMD) and age-matched healthy controls (HC)

Test	Eye		AMD patients		Healthy Controls		AMD Patients – Healthy Controls			AUC		
			Mean	SE	Mean	SE	Mean	Lower 95% CI		Mean	Lower 95% CI	Higher 95% CI
ACOLAPT	Worst Eye	PuAc	0.63	0.02	0.49	0.02	0.14	0.08	0.21	0.79	0.68	0.90
		PuCS	1.50	0.03	1.64	0.03	-0.14	-0.23	-0.05	0.67	0.53	0.80
		StCS	1.11	0.04	1.32	0.04	-0.21	-0.31	-0.09	0.76	0.64	0.88
		VaSt	3.43	0.33	5.18	0.33	-1.75	-2.69	-0.81	0.75	0.63	0.87
		∆CS	0.26	0.02	0.20	0.02	0.06	-0.00	0.13	0.70	0.56	0.83
		PuAc	0.56	0.02	0.49	0.02	0.06	0.01	0.12	0.67	0.54	0.81
	Best Eye	PuCS	1.56	0.04	1.63	0.04	-0.07	-0.20	0.05	0.61	0.47	0.74
		StCS	1.20	0.05	1.32	0.05	-0.12	-0.26	0.02	0.62	0.49	0.76
		VaSt	4.42	0.37	5.24	0.37	-0.82	-1.88	0.24	0.64	0.51	0.78
		∆CS	0.24	0.02	0.19	0.02	0.04	-0.02	0.11	0.62	0.47	0.76
OCT (ETDRS)	Worst Eye	CT	250.9	11.2	276.1	11.2	-25.2	-57.6	7.3	0.62	0.41	0.82
		ITT	320.8	5.1	327.9	5.1	-7.1	-21.9	7.7	0.59	0.39	0.79
		IST	330.7	5.1	338.0	5.1	-7.3	-21.9	7.3	0.64	0.44	0.85
		INT	329.7	6.2	339.0	6.2	-9.3	-27.2	8.6	0.65	0.45	0.86
		IIT	325.4	6.0	334.0	6.0	-8.5	-25.7	8.7	0.59	0.39	0.80
		OTT	282.3	3.5	285.4	3.5	-3.2	-13.1	6.8	0.59	0.36	0.82
		OST	298.1	4.6	298.9	4.6	-0.8	-14.1	12.4	0.59	0.38	0.79
		ONT	304.1	5.6	306.3	5.6	-2.2	-18.2	13.8	0.50	0.28	0.72
		OIT	283.4	4.2	283.0	4.2	0.4	-11.6	12.5	0.50	0.29	0.71
		Inner Mean	311.5	5.8	323.0	5.8	-11.5	-28.1	5.1	0.61	0.41	0.81
		Outer Mean	292.0	4.1	293.4	4.1	-1.4	-13.3	10.4	0.55	0.33	0.76
	Best Eye	CT	263.9	7.2	277.4	7.2	-13.5	-34.2	7.3	0.60	0.39	0.80
		ITT	317.4	5.3	326.2	5.3	-8.8	-24.2	6.6	0.63	0.43	0.83
		IST	332.0	4.9	336.8	4.9	-4.8	-19.0	9.4	0.61	0.40	0.82
		INT	334.6	5.6	338.1	5.6	-3.5	-19.6	12.7	0.58	0.37	0.79
		IIT	325.8	5.1	332.5	5.1	-6.7	-21.4	8.0	0.64	0.43	0.84
		OTT	280.2	3.3	282.0	3.3	-1.7	-11.1	7.6	0.59	0.37	0.81
		OST	295.6	3.5	297.6	3.5	-1.9	-12.0	8.2	0.56	0.34	0.77
		ONT	306.9	4.1	307.3	4.1	-0.4	-12.2	11.3	0.55	0.34	0.77
		OIT	281.5	3.5	282.1	3.5	-0.6	-10.8	9.5	0.58	0.36	0.79
		Inner Mean	314.8	5.2	322.2	5.2	-7.45	-22.4	7.5	0.61	0.40	0.81
		Outer Mean	291.1	3.3	292.2	3.3	-1.2	-10.8	8.5	0.59	0.38	0.81
Standard Tests	Worst Eye	IOP	14.0	0.9	13.9	0.9	0.1	-2.4	2.6	0.49	0.28	0.69
		Visual acuity	0.2	0.1	-0.1	0.05	0.3	0.1	0.4	0.83	0.69	0.97
		Contrast acuity	1.5	0.1	1.6	0.1	-0.1	-0.3	0.2	0.53	0.30	0.76
		Spherical	1.0	0.5	0.8	0.5	0.2	-1.2	1.5	0.50	0.29	0.71
		Cylindrical	-0.8	0.2	-0.9	0.2	0.1	-0.4	0.6	0.48	0.28	0.69
		Axis	88.7	12.7	78.9	12.7	9.8	-26.9	46.4	0.56	0.35	0.77
	Best Eye	IOP	14.0	0.8	14.0	0.8	-0.04	-2.4	2.3	0.47	0.26	0.67
		Visual acuity	0.0	0.03	-0.1	0.03	0.10	0.0	0.2	0.67	0.48	0.87
		Contrast acuity	1.6	0.05	1.7	0.05	-0.1	-0.2	0.1	0.62	0.40	0.84
		Spherical	0.8	0.4	1.0	0.4	-0.2	-1.4	1.1	0.54	0.34	0.75
		Cylindrical	-0.7	0.2	-0.9	0.2	0.20	-0.2	0.6	0.59	0.38	0.80
		Axis	89.8	12.4	60.3	12.4	29.5	-6.4	65.4	0.66	0.46	0.86

Mean values, standard error (SE), and 95% confidence intervals (CI) are presented together with receiver operating characteristic (ROC) analysis. PuAc = Pursuit acuity (BCAM “Objective Visual Acuity Test”); PuCS = Pursuit contrast sensitivity (BCAM “Objective Contrast Sensitivity Test”); StCS = Fixed-frequency, variable-contrast test; VaSt = Variable-frequency, fixed-contrast test; ΔCS = contrast sensitivity difference between the Fixed and Variable tests. OCT (ETDRS) values denote mean retinal thickness (µm) within the respective Early Treatment Diabetic Retinopathy Study (ETDRS) subfields; IOP = intraocular pressure

**Table 2:** Reliability of the Bulbicam ACOLAPT test variables

Eye	Variable	Day 1		Day 2		Day 2- Day 1			ICC	AI
		Mean	SE	Mean	SE	Mean	Lower 95% CI	Higher 95% CI		
Worst Eye	PuAc	0.65	0.03	0.62	0.03	-0.03	-0.13	0.06	0.83	0.75
	PuCS	1.51	0.05	1.51	0.05	0.00	-0.16	0.15	0.77	0.80
	StCS	1.11	0.06	1.12	0.06	0.01	-0.16	0.18	0.96	0.88
	VaSt	3.51	0.51	3.24	0.51	-0.27	-1.73	1.19	0.81	0.25
	∆CS	0.27	0.03	0.26	0.03	-0.01	-0.09	0.07	0.58	0.23
Best Eye	PuAc	0.56	0.03	0.57	0.03	0.01	-0.08	0.11	0.83	0.72
	PuCS	1.56	0.06	1.54	0.06	-0.03	-0.21	0.16	0.82	0.79
	StCS	1.15	0.08	1.22	0.08	0.07	-0.15	0.30	0.76	0.62
	VaSt	4.73	0.69	4.06	0.69	-0.67	-2.67	1.33	0.81	0.20
	∆CS	0.27	0.03	0.22	0.03	-0.05	-0.11	0.05	0.11	-0.48

The reliability is expressed by Intraclass Correlation (ICC) and the agreement within patients by Agreement Index (AI). The results are described by mean value, Standard Error (SE) and 95% confidence intervals. See Table 1 for ACOLAPT variable labels

**Table 3:** Stability of Bulbicam ACOLAPT test variables

Eye	Variable	M1	M2	M3	M4	M5	M6		SI			Classification	
									Mean	Lower 95% CI	Higher 95% CI	Population	Patient
Worst Eye (*N* = 17)	PuAc	0.65 (0.12)	0.62 (0.15)	0.63 (0.12)	0.63 (0.13)	0.61 (0.13)	0.63 (0.14)	0.62 (0.15)	0.56	0.56	0.71	+	+
	PuCS	1.51 (0.22)	1.53 (0.18)	1.50 (0.18)	1.50 (0.18)	1.49 (0.21)	1.51 (0.22)	1.53 (0.18)	0.48	0.48	0.69	+	+
	StCS	1.11 (0.24)	1.12 (0.23)	1.12 (0.29)	1.10 (0.25)	1.15 (0.24)	1.15 (0.31)	1.12 (0.23)	0.43	0.43	0.70	+	+
	VaSt	3.16 (1.85)	3.59 (2.22)	3.93 (2.71)	3.51 (1.99)	4.56 (2.30)	3.71 (2.03)	3.59 (2.22)	0.23	0.23	0.60	+	+
	∆CS	0.27 (0.11)	0.26 (0.11)	0.27 (0.13)	0.27 (0.14)	0.25 (0.16)	0.26 (0.15)	0.26 (0.11)	-0.00	-0.00	0.43	+	-
Best Eye (*N* = 17)	PuAc	0.56 (0.13)	0.57 (0.12)	0.59 (0.14)	0.60 (0.13)	0.59 (0.12)	0.56 (0.15)		0.61	0.54	0.69	+	+
	PuCS	1.55 (0.23)	1.55 (0.27)	1.56 (0.23)	1.52 (0.32)	1.56 (0.24)	1.53 (0.20)		0.62	0.52	0.73	-	-
	StCS	1.21 (0.29)	1.18 (0.29)	1.14 (0.30)	1.24 (0.24)	1.08 (0.38)	1.19 (0.25)		0.56	0.44	0.68	-	-
	VaSt	4.79 (3.14)	4.06 (2.53)	4.16 (1.80)	4.00 (1.84)	4.09 (2.16)	4.40 (2.49)		0.61	0.48	0.73	+	+
	∆CS	0.27 (0.18)	0.23 (0.07)	0.26 (0.14)	0.25 (0.17)	0.29 (0.16)	0.25 (0.10)		0.45	0.25	0.65	-	-

Six repeated measurements (M1–M6) are shown as mean values. Stability is expressed by the Stability Index (SI) with 95% confidence intervals (CI), and classification is based on SI thresholds. See Table 1 for ACOLAPT variable labels

#### Best eye

PuAc remained reduced in AMD (*p* = 0.026; Table 1). AUC was 0.67 (95% CI 0.53–0.80; Fig. 2a). ICC(3,1) was 0.83 (Table 2). AI was 0.72 (Fig. 3a). SI was 0.61 (95% CI 0.54–0.69), Excellent. Taken together, PuAc behaved consistently across eyes and qualified as a strong biomarker in both.

### Pursuit contrast sensitivity (PuCS)

#### Worst eye

PuCS was reduced in AMD (*p* = 0.04; Table 1). AUC was 0.67 (95% CI 0.53–0.80; Fig. 2b). ICC(3,1) was 0.76 (Table 2). AI was 0.80 (Fig. 3b). SI was 0.69, classified Excellent. PuCS therefore fulfilled validity and both repeatability domains and was classified as a strong biomarker.

#### Best eye

No difference was observed (*p* = 0.21; Table 1). AUC was 0.61 (95% CI 0.47–0.74; Fig. 2b). ICC(3,1) was 0.77 (Table 2). AI was 0.79 (Fig. 3b). SI was 0.62, Very Good. PuCS was therefore repeatable but not valid in the better eye and functioned as a physiological biomarker rather than a disease-discriminating measure.

### Strobe pursuit contrast sensitivity (StCS)

#### Worst eye

StCS was lower in AMD (*p* < 0.001; Table 1). AUC was 0.76 (95% CI 0.64–0.88; Fig. 2c). ICC(3,1) was 0.96 (Table 2). AI was 0.88 (Fig. 3c). SI was 0.43 (95% CI 0.43–0.70), classified Good. The variable met the validity and repeatability criteria and was therefore a strong biomarker.

#### Best eye

No significant difference was detected (*p* = 0.15; Table 1). AUC was 0.62 (95% CI 0.49–0.76; Fig. 2c). ICC(3,1) was 0.76 (Table 2). AI was 0.62 (Fig. 3c). SI was 0.56 (95% CI 0.44–0.68), Good–Very Good. In the better eye, StCS was repeatable but not valid and thus functioned as a physiological biomarker.

### Delta contrast sensitivity (ΔCS)

#### Worst eye

ΔCS was increased in AMD (*p* = 0.05; Table 1). AUC was 0.70 (95% CI 0.56–0.83; Fig. 2e), with the lower bound above 0.50, meeting the ROC validity criterion even though the AMD–HC confidence interval touched zero. ICC(3,1) was 0.58 (Table 2). AI was 0.23 (Fig. 3e). SI ranged from Poor to Good across eyes, with many below threshold (Table 3). ΔCS therefore met validity and between-patient repeatability but not within-patient repeatability, and was classified as a population biomarker only.

#### Best eye

ΔCS showed no group difference (*p* = 0.185; Table 1). AUC was 0.62 (95% CI 0.47–0.76; Fig. 2e), with the lower bound at chance. ICC(3,1) was 0.11 (Table 2). AI was − 0.48 (Fig. 3e). SI was 0.45 (Table 3). ΔCS was therefore neither valid nor repeatable and did not qualify as a biomarker in the better eye.

### Variable Strobe Pursuit Object Detection (VaSt)

#### Worst eye

**Fig. 4:**
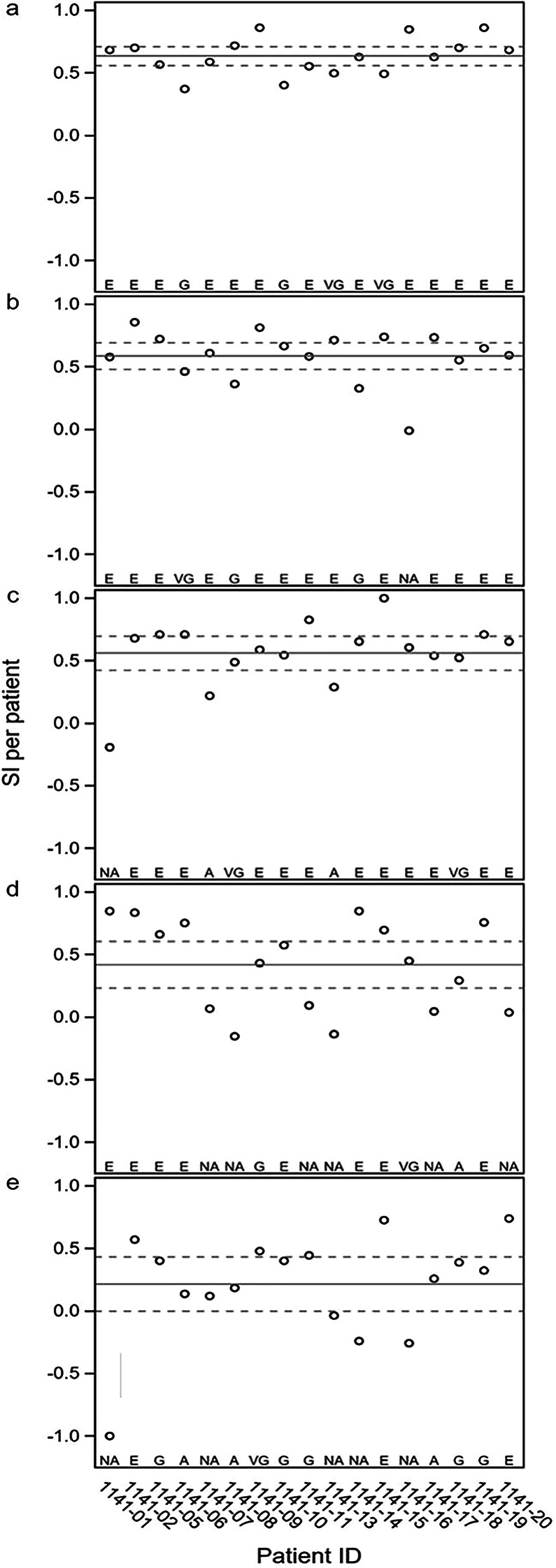
Individual Stability Index (SI) of the worst eye for all included patients are given for **a**) Pursuit acuity (PuAc); **b**) Pursuit contrast sensitivity (PuCS); **c**) Fixed frequency Variable contrast strobe (StCS); **d**) Variable frequency Fixed contrast strobe (VaSt), and **e**) ∆CS. The horizontal line shows the mean SI and the dotted line shows the 95% confidence interval. The participating patients are indicated on the x-axis with the classification symbols E=excellent, VG=very good, G=good, A= acceptable and NA = not acceptable **a**) Pursuit acuity (PuAc); **b**) Contrast Sensitivity (CS); **c**) Fixed frequency, variable contrast strobe (StCS); **d**) Variable frequency, fixed contrast strobe (VaSt) and **e**) Delta contrast sensitivity (∆CS)

VaSt was lower in AMD (*p* < 0.001; Table 1). AUC was 0.76 (95% CI 0.64–0.88; Fig. 2d). ICC(3,1) was 0.81 (Table 2). AI was 0.25 (Fig. 3d). SI ranged from 0.23 to 0.61 (Table 3), with most AMD eyes classified as Good or Acceptable (Fig. 4d). The AI–SI rescue rule was met, and the variable remained valid and repeatable across both domains. VaSt was therefore classified as a strong biomarker in the worst eye.

#### Best eye

No significant group difference was observed (*p* = 0.135; Table 1). AUC was 0.64 (95% CI 0.51–0.78; Fig. 2d). ICC(3,1) was 0.81 (Table 2). AI was 0.20 (Fig. 3d). SI was 0.61 (95% CI 0.48–0.73; Table 3), satisfying the AI–SI rescue rule. The lower AUC confidence bound exceeded 0.50, indicating validity despite a modest effect size. VaSt therefore qualified as a strong biomarker in both eyes, though with lower precision than the other ACOLAPT variables.

### Extended profiles of the candidate biomarkers

Using the predefined rules, PuAc, PuCS, StCS and VaSt were all strong biomarkers in the worst eye, each meeting validity and both repeatability domains, the latter through the AI–SI rescue rule for VaSt. ΔCS also met validity and between-patient repeatability in the worst eye but lacked within-eye stability and was therefore a population biomarker only. None of the reviewed Nidek RS-3000 OCT variables achieved validity.

Across both eyes, PuAc showed the most consistent profile, remaining valid and repeatable in the better eye. PuCS and StCS retained high repeatability in the better eye but did not discriminate AMD, functioning therefore as physiological biomarkers. VaSt, strong in the worst eye, also met validity and the AI–SI rescue rule together with acceptable between-patient repeatability in the better eye, and was therefore strong in both eyes. ΔCS was neither valid nor repeatable in the better eye.

Taken together, ACOLAPT identified both population- and patient-level biomarkers within a single functional domain, with strongest performance in the worse eye where disease-related variation was greatest. No OCT-derived variable distinguished AMD from controls or separated best from worst eyes. Table 3 summarises these classifications.

A full statistical narrative is provided in Supplement 3 (Extended Statistical Results Narrative) to examine each variable using standard ANOVA, ROC, and repeatability conventions.

## Discussion

### Clinical interpretation

ACOLAPT detected functional disturbance in dry AMD at a stage when OCT thickness remained within known normal limits, and when chart measures showed only modest separation. The five subtests examined complementary aspects of cone-driven function and produced a coherent sequence of change across the better and worse eyes, consistent with dynamic disturbance emerging before measurable structural deviation [5, 21, 22].

#### Better eye

In the better eye, structural signs of AMD were limited or entirely absent in several participants, yet two ACOLAPT variables demonstrated reliable separation. PuAc showed a small but reproducible difference between AMD and controls, supported by an AUC of 0.67 and strong within-eye repeatability. Chart acuity revealed a parallel difference, although with weaker consistency, in keeping within the known variability of high-contrast logMAR testing in early disease. Dynamic spatial precision therefore resolved early disturbance with greater stability than static charts in this cohort [23].

VaSt also distinguished AMD from controls even when Pelli–Robson contrast remained within normal limits. This pattern is consistent with broader visual science literature showing that temporal contrast mechanisms are often more vulnerable than static contrast measures, and that Pelli–Robson thresholds may remain normal despite measurable losses in temporal modulation sensitivity. PuCS and StCS showed no separation in the better eye, suggesting that steady contrast gain control remains preserved at this stage.

#### Worse eye

In the worse eye, four ACOLAPT variables showed clear separation between groups. PuAc differences were larger, while PuCS and StCS, which were preserved in the better eye, now diverged. VaSt showed the largest temporal shift. ΔCS reached population-level validity but lacked within-eye precision. Chart acuity and Pelli–Robson contrast decreased but with broader overlap between groups than the dynamic measures. These findings suggest that spatial precision, steady contrast gain, temporal recovery, and temporal endurance are all reduced in more affected eyes, consistent with a shared vulnerability as functional stress accumulates.

#### Integrated functional sequence

Combining ACOLAPT with chart measures suggests a plausible sequence of events. In the better eye, dynamic spatial precision and temporal endurance diverge first, while chart acuity shows only a small shift and Pelli–Robson contrast remains stable. As dysfunction progresses, steady contrast mechanisms begin to fail, producing differences in PuCS and StCS. Pelli–Robson contrast falls at a later point, with substantial overlap between groups, consistent with earlier reports [24]. In the worse eye, four ACOLAPT variables separate robustly while conventional OCT thickness metrics show only modest, heterogeneous deviations from published values for early and intermediate AMD [25]. This is compatible with rapid adaptive processes changing before thickness measures diverge.

### Practical implications for clinicians

In clinical settings, ACOLAPT is most informative when chart acuity is normal or near-normal but patients describe intermittent blur, reduced CS, slower reading, or delayed recovery after exposure to bright scenes. It may also be helpful when OCT shows minimal structural change, or when following functional progression between routine imaging visits. High-stability variables such as PuAc, PuCS, and StCS appear to be well suited for monitoring and screening, subject to further validation. Temporal measures such as VaSt and ΔCS have a lower AI, but moderate SI, and therefore require averaging of repeated trials but quantify a property that classical charts cannot measure.

### Relevance for clinical trials

Because ACOLAPT provides operator-independent thresholds, demonstrates stable within-eye metrics across sessions, and detects small deviations in adaptable cone function, it may support early-phase therapeutic studies. The stability indices suggest potential utility as exploratory or secondary endpoints, particularly for interventions aimed at early metabolic or synaptic stress.

### Mechanistic interpretation

The graded pattern is consistent with models where metabolic and microstructural strain in the cone–RPE complex first affects rapid adaptive processes. Early shifts in dynamic acuity and temporal endurance align with reduced metabolic resilience rather than overt structural loss, although direct causal inference cannot be made here. As functional reserve declines, post-receptoral gain control appears less stable, affecting both steady contrast sensitivity and recovery after transient suppression. Histological studies describe early outer plexiform reorganisation and Müller-cell engagement in eyes that appear structurally unremarkable on OCT, providing a possible context for these observations. The later rise in ΔCS may mirror the increasing cost of temporal modulation once adaptive reserve is strained [26–28].

### Interpretation of variability

Higher intra-subject variability in VaSt and ΔCS among AMD participants might reflect biological instability rather than measurement noise. Experimental and imaging studies report mitochondrial fragmentation, reduced choriocapillaris flow, and early metabolic inefficiency in eyes that remain structurally normal on OCT. Reduced energetic margin may produce unstable recovery between flicker pulses, an effect that carries diagnostic meaning rather than noise .

### Learning effects

We found no clear evidence of systematic learning across sessions. Mean differences in repeated measurements as demonstrated by Bland-Altman plots showed no directional trend and remained within the range expected for test–retest variability in short adaptive psychophysical tasks [Baker et al. 2008].

### Adaptation without pigment bleaching

Stimuli were kept below the photopigment bleaching threshold to ensure that performance reflected neural adaptation rather than pigment depletion. This approach is intended to emphasise processes such as calcium feedback, cGMP turnover, and retinal gain control [Pugh and Lamb 2000]. Future work can extend the protocol into the bleaching range to examine transitions from neural adaptation to photochemical recovery across the photopic to mesopic continuum.

### Reliability and interpretive logic

Reliability was examined at complementary levels following the scheme in Supplement 1. ICC quantified relative consistency across participants but was interpreted with caution because ICC increases when between-participant variance exceeds within-eye noise [Koo and Li 2016]. AI quantified within-eye precision and therefore defined suitability for single-eye staging. SI assessed steadiness across sessions and indicated whether a variable can support longitudinal monitoring.

PuAc and PuCS reached high ICC, AI, and SI, supporting their suitability for both population-level research and patient-level follow-up. StCS achieved the same standard in the worse eye. VaSt and ΔCS showed low AI but moderate SI, suggesting that single-trial estimates are noisy but that pooled or averaged measurements across trials remain stable. High SI with modest AI is therefore compatible with longitudinal monitoring, whereas low SI restricts suitability for single-trial screening.

Because ICC alone cannot determine clinical repeatability, all conclusions regarding patient-level use rely on AI and SI rather than population-level consistency. This distinction prevents inflation of reliability estimates in metrics with high between-participant variance.

### Clinical summary

PuAc and VaSt detected early functional disturbance with greater stability than chart tests in this study. In the worse eye, four variables met criteria for both population and patient-level use. ΔCS provided population-level discrimination but lacked the precision required for individual monitoring. Charts retained clinical value but did not detect early disturbance with comparable sensitivity. Taken together, these variables quantify a hierarchy of cone-driven dysfunction that appears to precede detectable structural change in this cohort.

### Limitations

This study has several limitations. Participants were recruited through clinic-based and public advertisement, which may introduce selection bias and limit generalisability. The sample size was modest and drawn from a single centre using a single device. Repetition counts differed between eyes and groups. VaSt and ΔCS showed lower within-eye agreement. Physiological interpretations are preliminary and based on consistency with published models rather than direct evidence. OCT thickness is a coarse comparator that may not reflect early metabolic or synaptic stress.

### Future directions

Future studies should test ACOLAPT in larger cohorts, evaluate its behaviour across AMD subtypes, compare it directly with dark adaptation protocols, and examine links with metabolic imaging markers such as fundus autofluorescence or choriocapillaris flow.

The short test duration, automated thresholding, and stable cross-session behaviour suggest readiness for multi-centre evaluation. 

## Supplementary Information

Below is the link to the electronic supplementary material.

Supplementary Material 1

## Data Availability

Source data have been secured in a locked database, courtesy of Meddoc research. Data will be made available upon request.

## References

[1] Bourne RRA, Steinmetz JD, Saylan M, et al. Causes of blindness and vision impairment in 2020 and trends over 30 years, and prevalence of avoidable blindness in relation to VISION 2020: the Right to Sight: an analysis for the Global Burden of Disease Study. Lancet Glob Health. 2020;9:e144.33275949

[2] Lim LS, Mitchell P, Seddon JM, Holz FG, Wong TY. Age-related macular degeneration. Lancet. 2012;379:1728–38. 22559899 10.1016/S0140-6736(12)60282-7

[3] Ambati J, Fowler BJ. Mechanisms of age-related macular degeneration. Neuron. 2012;75:26–39. 22794258 10.1016/j.neuron.2012.06.018 PMC3404137

[4] Johnson PT, Brown MN, Pulliam BC, Anderson DH, Johnson LV. Synaptic Pathology, Altered Gene Expression, and Degeneration in Photoreceptors Impacted by Drusen. Invest Ophthalmol Vis Sci. 2005;46:4788–95. 16303980 10.1167/iovs.05-0767

[5] Spaide RF, Curcio CA, ANATOMICAL CORRELATES TO THE BANDS SEEN IN THE OUTER RETINA BY OPTICAL COHERENCE TOMOGRAPHY. Literature Rev Model Retina. 2011;31:1609. 10.1097/IAE.0b013e3182247535 PMC3619110 21844839

[6] Johnson PT, Brown MN, Pulliam BC, Anderson DH, Johnson LV. Synaptic pathology, altered gene expression, and degeneration in photoreceptors impacted by drusen. Invest Ophthalmol Vis Sci. 2005;46:4788–95. 16303980 10.1167/iovs.05-0767

[7] Ţurcaş C, Nicoară SD. A comprehensive review of structure-function correlations in age-related macular degeneration: Contributions of microperimetry. Surv Ophthalmol. 2025;70:426–50. 39828006 10.1016/j.survophthal.2025.01.009

[8] Sun H, Swanson WH, Arvidson B, Dul MW. Assessment of contrast gain signature in inferred magnocellular and parvocellular pathways in patients with glaucoma. Vis Res. 2008;48:2633–41. 18501947 10.1016/j.visres.2008.04.008 PMC2825154

[9] Pokorny J. Review: Steady and pulsed pedestals, the how and why of post-receptoral pathway separation. J Vis. 2011;11:7–7. 10.1167/11.5.7 21737512

[10] Beaudoin DL, Borghuis BG, Demb JB. Cellular basis for contrast gain control over the receptive field center of mammalian retinal ganglion cells. J Neurosci. 2007;27:2636–45. 17344401 10.1523/JNEUROSCI.4610-06.2007 PMC6672510

[11] Lucassen M, Lambooij M, Sekulovski D, Vogels I. Spatio-chromatic sensitivity explained by post-receptoral contrast. J Vis. 2018. 10.1167/18.5.13. 29904788 10.1167/18.5.13

[12] Lad EM, Finger RP, Guymer R. Biomarkers for the Progression of Intermediate Age-Related Macular Degeneration. Ophthalmol Ther. 2023;12:2917. 37773477 10.1007/s40123-023-00807-9 PMC10640447

[13] Dunn FA, Lankheet MJ, Rieke F. Light adaptation in cone vision involves switching between receptor and post-receptor sites. Nature. 2007;449:603–6. 17851533 10.1038/nature06150

[14] Jackson GR, Scott IU, Kim IK, Quillen DA, Iannaccone A, Edwards JG. Diagnostic sensitivity and specificity of dark adaptometry for detection of age-related macular degeneration. Invest Ophthalmol Vis Sci. 2014;55:1427–31. 24550363 10.1167/iovs.13-13745 PMC3954002

[15] Guymer RH, Tan RS, Luu CD. Comparison of Visual Function Tests in Intermediate Age-Related Macular Degeneration. Transl Vis Sci Technol. 2021;10:14. 34636906 10.1167/tvst.10.12.14 PMC8525848

[16] Pilotto E, Convento E, Guidolin F, Abalsamo CK, Longhin E, Parrozzani R, Midena E. Microperimetry Features of Geographic Atrophy Identified With En Face Optical Coherence Tomography. JAMA Ophthalmol. 2016;134:873. 27253760 10.1001/jamaophthalmol.2016.1535

[17] Vujosevic S, Pucci P, Casciano M, Longhin E, Convento E, Bini S, Midena E. Long-term longitudinal modifications in mesopic microperimetry in early and intermediate age-related macular degeneration. Graefe’s Archive Clin Experimental Ophthalmol. 2017;255:301–9. 10.1007/s00417-016-3466-z 27582087

[18] Koo TK, Li MY. A Guideline of Selecting and Reporting Intraclass Correlation Coefficients for Reliability Research. J Chiropr Med. 2016;15:155–63. 27330520 10.1016/j.jcm.2016.02.012 PMC4913118

[19] Pugh EN, Lamb TD. Phototransduction in vertebrate rods and cones molecular mechanisms of amplification, recovery and light adaptation.

[20] National Institute for Health and Care Excellence (NICE). (2018) Age-related macular degeneration: diagnosis and management. London. 29400919

[21] Owsley C. Contrast sensitivity. Ophthalmol Clin North Am. 2003;16:171–7. 12809156 10.1016/s0896-1549(03)00003-8

[22] Pelli DG, Bex P. Measuring contrast sensitivity. Vis Res. 2013;90:10–4. 23643905 10.1016/j.visres.2013.04.015 PMC3744596

[23] Rosser DA, Laidlaw AH. Murdoch IE. The development of a reduced logMAR visual acuity chart for use in routine clinical practice. 10.1136/bjo.85.4.432 PMC1723918 11264133

[24] Rubin GS, West SK, SEE Project Team. A Comprehensive Assessment of Visual Impairment in a Population of Older Americans. Invest Ophthalmol Vis Sci. 1997;38:557–68. 9071208

[25] Wood A, Binns A, Margrain T, Drexler W, Považay B, Esmaeelpour M, Sheen N. Retinal and Choroidal Thickness in Early Age-Related Macular Degeneration. Am J Ophthalmol. 2011;152:1030–e10382. 21851922 10.1016/j.ajo.2011.05.021

[26] Wong JHC, Ma JYW, Jobling AI, Brandli A, Greferath U, Fletcher EL, Vessey KA. Exploring the pathogenesis of age-related macular degeneration: A review of the interplay between retinal pigment epithelium dysfunction and the innate immune system. Front Neurosci. 2022. 10.3389/fnins.2022.1009599. 36408381 10.3389/fnins.2022.1009599 PMC9670140

[27] Owsley C, Huisingh C, Clark ME, Jackson GR, McGwin G. Comparison of Visual Function in Older Eyes in the Earliest Stages of Age-related Macular Degeneration to Those in Normal Macular Health. Curr Eye Res. 2016;41:266–72. 25802989 10.3109/02713683.2015.1011282 PMC4737986

[28] Gillespie-Gallery H. Spatial and temporal aspects of visual performance in relation to light level and normal aging.

