# Supplementary material for: Eye-tracking-derived foveal biomarkers and functional alterations in dry AMD: findings from a controlled clinical study
Source: Int J Retina Vitreous. 2026 Mar 27;12:70. doi: 10.1186/s40942-026-00838-x (PMC13147856; doi:10.1186/s40942-026-00838-x)
Supplement: Supplementary file 1 — Supplementary Material 1 [file 40942_2026_838_MOESM1_ESM.docx]

**Supplementary Material**

*Eye-Tracking-Derived Foveal Biomarkers and Functional Alterations in Dry AMD*

**Abbreviations:** AI = agreement index; AMD = age-related macular degeneration; ANOVA = analysis of variance; AUC = area under the curve; CI = confidence interval; HC = healthy control; ICC = intraclass correlation coefficient; ROC = receiver operating characteristic; SI = stability index.

# Supplement 1. Biomarker Decision Rules

We classified variables according to the following framework:

## Validity

Validity was defined as discrimination between AMD and HC, demonstrated if either

1. the ROC AUC lower 95% CI exceeded 0.50, or
2. the AMD–HC confidence interval excluded zero (equivalent to *p* < 0.05 in ANOVA).

In practice, both ROC and ANOVA were reported for each variable to provide concordant evidence, but either criterion alone was sufficient to establish validity.

## Repeatability

***Between patients (population level)***

- ICC (v.3.1) > 0.50

***Within patients (individual level)***

- AI > 0.50 and SI > 0.14
  - **Exception A (rescue):** if AI ≤ 0.50 but SI > 0.14, the variable was still accepted as within-patient repeatable.
  - **Exception B:** if AI > 0.50 but SI ≤ 0.14, the variable was considered repeatable but flagged as not stable.

## Stability

SI > 0.14 → stable. Stability was evaluated independently of validity and discrimination. A variable could therefore be stable even if it was not valid as a biomarker.

## Reliability

Reliability refers solely to the behaviour of the measurement itself. A variable was considered reliable if it met both repeatability criteria: between-patient repeatability (ICC > 0.50) and within-patient repeatability according to the AI/SI rules. SI functions as a stability qualifier rather than a general requirement and is invoked where across-session stability bears on the intended clinical use. Reliability therefore reflects the reproducibility of the physiological signal, independent of whether the variable distinguishes AMD from healthy controls.

## Biomarker Classification

In this framework, we distinguish between *physiological biomarkers* and *disease-discriminating biomarkers*. Physiological biomarkers reflect stable and quantifiable aspects of visual function, independent of whether they separate AMD from healthy controls. Disease-discriminating biomarkers require both validity and repeatability criteria.

| **Classification** | **Criteria** |
| --- | --- |
| Physiological biomarker | Acceptable repeatability (population, within-patient, or both); no validity requirement |
| Reliable biomarker | Acceptable repeatability at both population and within-patient level |
| Disease-discriminating biomarker | Validity criterion met (ROC AUC lower 95% CI > 0.50 or AMD–HC CI excludes zero) |
| Population biomarker | Valid + ICC > 0.50 |
| Patient biomarker | Valid + within-patient repeatability (AI/SI rules) |
| Strong biomarker | Valid + reliable (both repeatability domains) |

## Interpretation

- Variables meeting both repeatability domains (ICC > 0.50 and within-patient repeatability per AI/SI rules) were considered **reliable** measures of the underlying physiological process. Reliable measures can function as physiological biomarkers irrespective of whether they discriminate between AMD and HC.
- Variables with only between-patient repeatability (ICC > 0.50, AI/SI not satisfied) were considered useful for **population-level description** of visual function. If they also met the validity criterion, they were classified as **population biomarkers**; otherwise they were regarded as population-level physiological measures.
- Variables with only within-patient repeatability and stability (AI/SI rules satisfied, ICC ≤ 0.50) were considered suitable for **individual clinical assessment**. If they also met the validity criterion, they were classified as **patient biomarkers**; otherwise they were regarded as within-patient physiological measures.
- Variables fulfilling validity and both repeatability domains were classified as **strong biomarkers**, applicable in both population research and individual patient monitoring.
- **Stability** (SI > 0.14) added confidence for longitudinal use but was not treated as a formal requirement for validity in this initial evaluation.

# Supplement 2. Statistical Methodology

*Note:* This section is adapted with permission from Dalbro et al (2025)^1^. The original author has reviewed and consented to the use and minor modification of this text.

## Overview and Rationale

This study distinguishes between **population-level reproducibility** and individual-level repeatability (same-eye, same-condition) and temporal stability (same-eye, cross-session).

The *Intraclass Correlation Coefficient* (ICC, model 3.1) quantifies repeatability across participants, while the *Bland–Altman* framework, the *Agreement Index* (AI), and the *Stability Index* (SI) describe within-participant agreement and temporal stability.

Population-level reliability considers both inter- and intra-participant variance, which is useful for validating a measurement as a research instrument.

Clinical reliability, however, depends on how reproducible a result is within a single participant. Because ICC incorporates between-participant heterogeneity, it may appear high even when individual measurements vary widely. ICC should therefore be regarded as a research metric, not a clinical one, unless supported by within-participant indices^2^.

## Population Reliability – Intraclass Correlation Coefficient (ICC)

Population reliability was estimated using the **two-way mixed-effects, absolute-agreement model ICC(3,1)** [30]. This model assesses the degree of absolute agreement rather than repeatability alone and is suitable for fixed raters or instruments.

Categorisation followed established guidelines^2,3^:

| **Category** | **ICC Range** |
| --- | --- |
| Poor | < 0.50 |
| Moderate | [0.50, 0.75) |
| Good | [0.75, 0.90) |
| Excellent | [0.90, 1.00] |

Confidence intervals for ICC(3,1) were calculated using standard parametric methods [30].

## Within-Participant Agreement – Bland–Altman Model and Agreement Index (AI)

Within-participant repeatability was analysed using the **Bland–Altman method^4,5^**.

For two repeated measures on the same eye, the mean difference and the 95% limits of agreement (LoA) were computed as:

*Mean difference ± 2 × SD_diff_*

where *SD*_diff_ is the standard deviation of pairwise differences between the two observations. These limits represent the interval in which 95% of repeated differences are expected to fall.

To obtain a scale-free summary of agreement, we calculated the **Agreement Index (AI)** (Suther et al., 2018), defined as:

$$AI=1-\frac{2\times{SD}_{w}}{Mean level of the measurements}$$

where *SD*_w_ is the within-subject standard deviation. AI increases as the LoA narrow relative to the mean value.

The formula is algebraically equivalent to

$$AI=1-\frac{2\times\mathrm{SD}_{\mathrm{diff}}}{\mathrm{mean}}$$

Since

$${SD}_{w}=\frac{\mathrm{SD}_{\mathrm{diff}}}{\sqrt{2}}$$

when two repeats are compared.

AI values were categorised identically to ICC (see table above).

## Across-Session Stability – Stability Index (SI)

Let *SD*_w_ be the standard deviation within patients and *SD*_b_ the standard deviation between patients. The ratio SD_w_/SD_b_ indicates stability: a smaller ratio implies greater stability.

To make the index increase with stability, we define:

*SI = 1 – (SD_w_ / SD_b_)*

Ratios < 1 imply acceptable stability.

## Probability-Based Classification

Unlike arbitrary cut-offs, our limits derive from the F-distribution of variance ratios.

Let *n* denote the number of repeated observations per patient and *m* the number of patients. The statistic:

$$F \{[{S_{w}}^{2}/{S_{b}}^{2}]\times[(m-1)/(n-1)]\}=$$

$$F \{[{S_{w}}^{2}/(n-1)]/[{S_{b}}^{2}/(m-1)]\}=$$

$$F \{{[{SD}_{w}/{SD}_{b}]}^{2}\}$$

follows an F-distribution with (*n* – 1, *m* – 1) degrees of freedom^6^.

If *f*_1–α_ is the (1 – α) quantile of this F-distribution, then:

$$P \left[ {({SD}_{w}/{SD}_{b})}^{2}\leq f_{1-\alpha} \right]=1-\alpha\Longrightarrow P \left[ \left( {SD}_{w}/{SD}_{b} \right)\leq\sqrt{f_{1-\alpha}} \right]=1-\alpha$$

Thus, α determines the classification thresholds for SI.

With *n* = 6 repeats and *m* = 16 participants, (SD_w_/SD_b_)² ~ F_(5,15)_.

Using α = 0.05, 0.10, 0.20, 0.40 gives the following SI classification thresholds (see Supplementary Table 1 for calculation):

| **Category** | **SI Range** |
| --- | --- |
| Excellent | ≥ 0.53 |
| Very Good | [0.44, 0.53) |
| Good | [0.34, 0.44) |
| Acceptable | [0.14, 0.34) |
| Poor | < 0.14 |

These classifications apply both to each patient’s SI and to the mean SI of the cohort.

Confidence intervals for SI were estimated by non-parametric bootstrap (10,000 resamples per subtest and eye).

## Workflow and Unit of Analysis

ICC(3,1) quantified population-level reliability across AMD and control cohorts. AI and SI were computed per eye to assess within-session agreement and across-session stability.

## Software and Reproducibility

All analyses were performed in **SAS 9.04 Maintenance release M6**. Time-stamped logs, intermediate tables, and computational scripts are archived and available on reasonable request.

# Supplement 3. Extended Statistical Results Narrative

This appendix provides the extended analytic summaries prepared during internal statistical review. All results correspond to the values reported in Tables 1–3 and Figures 1–4 of the main manuscript.

Variable names have been standardised to match ACOLAPT nomenclature:

| **Abbreviation** | **Full Name** |
| --- | --- |
| **PuAc** | Pursuit acuity |
| **PuCS** | Pursuit contrast sensitivity |
| **StCS** | Fixed-frequency, variable-contrast |
| **VaSt** | Variable-frequency, fixed-contrast |
| **ΔCS** | Difference between fixed-contrast and variable-contrast thresholds |

## Extended Results Narrative

***Pursuit Acuity (PuAc)***

PuAc was significantly elevated (worse) in AMD compared with healthy controls in both eyes.

**Worst eye.** *p* ≤ 0.001 (Table I). ROC AUC was significantly above chance (AUC > 0.50; *p* ≤ 0.05) (Fig. 1a). The standard chart test (SCT) also detected a significant difference (*p* < 0.01), but only in the worst eye. BCAM and SCT did not differ significantly in their measured thresholds (Fig. 2a), and a positive correlation was observed.

- *Reliability analysis.* ICC indicated good reliability (Table II). AI showed good agreement in the worst eye (Fig. 3a).
- *SI* classified overall stability as Excellent for both eyes (Table III), with only one patient classified as Very Good (Fig. 4a).

**Best eye.** *p* ≤ 0.04 (Table I). ROC AUC remained significantly above 0.50 (Fig. 1a). ICC indicated good reliability, AI indicated moderate agreement (Fig. 3b), and SI again showed Excellent stability (Table III).

***Pursuit Contrast Sensitivity (PuCS)***

**Worst eye.** PuCS was significantly reduced in AMD (*p* = 0.04; Table I). ROC AUC was significantly above 0.50 (*p* = 0.03; Fig. 1b). The SCT did not detect a significant difference, though ROC analysis showed a borderline BCAM > SCT trend (*p* = 0.06; Fig. 2b). A slight positive correlation between BCAM and SCT was observed.

- Reliability analysis: ICC classified reliability as good (Table II).
- AI indicated good agreement for both eyes (Fig. 3c,d).
- SI was Excellent for both eyes (Table III), with individual SI: Excellent (13 participants), Very Good (1), Good (2), Not Acceptable (1) (Fig. 4b).
- **Best eye:** no significant difference detected (Table I; Fig. 1b).

***StCS (Fixed Frequency, Variable Contrast)***

**Worst eye:** significant reduction in AMD (*p* = 0.03; Table I). ROC AUC significantly exceeded 0.50 (*p* = 0.02; Fig. 1c).

**Reliability analysis:**

- ICC = Excellent on the worst eye, Good on the best (Table II)
- AI = Good on the worst eye, Moderate on the best (Fig. 3e,f)
- SI = Excellent overall for both eyes (Table III)
- Individual SI classifications (Fig. 4c): Excellent (13), Very Good (1), Acceptable (2), Not Acceptable (1)

**Best eye.** No significant AMD–HC difference (Table I).

***VaSt (Variable Frequency, Fixed Contrast)***

**Worst eye.** Significantly reduced (*p* = 0.03; Table I).

- ROC AUC was above chance for both eyes (*p* ≤ 0.05; Fig. 1d).
- ICC indicated good reliability for both eyes (Table II).
- AI classified poor agreement in both eyes (Fig. 3g,h).
- SI was Very Good on the worst eye and Excellent on the best (Table III).
- Individual SI (Fig. 4d): Excellent (9), Very Good (1), Good (1), Acceptable (4), Not Acceptable (2).

**Best eye.** No significant mean difference by ANOVA, but ROC AUC remained significantly above 0.50 (Fig. 1d).

***Delta Contrast Sensitivity (ΔCS)***

**Worst eye.** Significantly reduced in AMD (*p* = 0.05; Table I).

- ROC AUC was significantly above chance (*p* = 0.03; Fig. 1e).
- ICC = moderate reliability on the worst eye, poor on the best (Table II).
- AI = poor agreement for both eyes (Fig. 3i,j).
- SI = Acceptable on the worst eye and Very Good on the best (Table III).
- Individual SI distribution (Fig. 4e): Excellent (3), Very Good (2), Good (3), Acceptable (3), Not Acceptable (6).

**Best eye.** No significant difference (*p* = 0.185; Table I) and ROC AUC not above chance (Fig. 1e).

**Interpretation Note:** *The extended statistical narrative confirms that the conclusions of the main manuscript are robust to standard ANOVA–ROC reporting and that no classification outcome changes under the alternative phrasing used in internal review. All values match those in Tables 1–3 and Figures 1–4.*

# References

1. Dalbro, S. E. J. *et al.* Repeatability, reliability, and stability of eye movement measurements in Parkinson’s disease, cerebellar ataxia, and healthy adults. *Front. Neurol.* **16**, (2025).

2. Koo, T. K. & Li, M. Y. A Guideline of Selecting and Reporting Intraclass Correlation Coefficients for Reliability Research. *J. Chiropr. Med.* **15**, 155–163 (2016).

3. Aarås, A., Veierød, M. B., Larsen, S., Ørtengren, R. & Ro, O. Reproducibility and stability of normalized EMG measurements on musculus trapezius. *Ergonomics* **39**, 171–185 (1996).

4. Bland, M. J. & Altman, D. G. Statistical methods for assessing agreement between two methods of clinical measurement. *The Lancet* **327**, 307–310 (1986).

5. Bland, J. M. & Altman, D. G. Applying the right statistics: Analyses of measurement studies. *Ultrasound in Obstetrics and Gynecology* vol. 22 85–93 Preprint at https://doi.org/10.1002/uog.122 (2003).

6. Bland, M. *An Introduction to Medical Statistics*. (Oxford University Press, 1995).
